# Matrix metalloproteinase-10 promotes tumor progression through regulation of angiogenic and apoptotic pathways in cervical tumors

**DOI:** 10.1186/1471-2407-14-310

**Published:** 2014-05-03

**Authors:** Ge Zhang, Makito Miyake, Adrienne Lawton, Steve Goodison, Charles J Rosser

**Affiliations:** 1 Cancer Research Institute, MD Anderson Cancer Center Orlando, Orlando Health, Orlando, FL 32827, USA; 2 Department of Pathology, Orlando Health, Orlando, FL 32806, USA; 3 Nonagen Bioscience Corporation, Orlando, FL 32827, USA; 4 Department of Health Sciences Research, Mayo Clinic, Jacksonville, FL, USA; 5 Clinical and Translational Research Program, University of Hawaii Cancer Center, Ilalo Street #327, Honolulu, HI 96813, USA

**Keywords:** Angiogenesis, Apoptosis, Cancer, Invasion, MMP-10

## Abstract

**Background:**

Cancer invasion and metastasis develops through a series of steps that involve the loss of cell to cell and cell to matrix adhesion, degradation of extracellular matrix and induction of angiogenesis. Different protease systems (*e.g.,* matrix metalloproteinases, MMPs) are involved in these steps. MMP-10, one of the lesser studied MMPs, is limited to epithelial cells and can facilitate tumor cell invasion by targeting collagen, elastin and laminin. Enhanced MMP-10 expression has been linked to poor clinical prognosis in some cancers, however, mechanisms underlying a role for MMP-10 in tumorigenesis and progression remain largely unknown. Here, we report that MMP-10 expression is positively correlated with the invasiveness of human cervical and bladder cancers.

**Methods:**

Using commercial tissue microarray (TMA) of cervical and bladder tissues, MMP-10 immunohistochemical staining was performed. Furthermore using a panel of human cells (HeLa and UROtsa), *in vitro* and *in vivo* experiments were performed in which MMP-10 was overexpressed or silenced and we noted phenotypic and genotypic changes.

**Results:**

Experimentally, we showed that MMP-10 can regulate tumor cell migration and invasion, and endothelial cell tube formation, and that MMP-10 effects are associated with a resistance to apoptosis. Further investigation revealed that increasing MMP-10 expression stimulates the expression of HIF-1α and MMP-2 (pro-angiogenic factors) and PAI-1 and CXCR2 (pro-metastatic factors), and accordingly, targeting MMP-10 with siRNA *in vivo* resulted in diminution of xenograft tumor growth with a concomitant reduction of angiogenesis and a stimulation of apoptosis.

**Conclusion:**

Taken together, our findings show that MMP-10 can play a significant role in tumor growth and progression, and that MMP-10 perturbation may represent a rational strategy for cancer treatment.

## Background

The extracellular microenvironment is an integral and dynamic scaffold that dictates cell function and cell fate for both normal and cancer cells. Cancer invasion and metastasis develop through a series of steps that involve the loss of cell to cell and cell to matrix adhesion, degradation of extracellular matrix and induction of angiogenesis. A number of protease systems, including the matrix metalloproteinases (MMPs) are involved in these steps. MMPs are essential regulators of the tissue microenvironment through their ability to control extracellular matrix (ECM) turnover [1]. The MMP family is comprised of at least 24 members that can collectively process virtually any component of the ECM [2], thereby influencing basic cellular processes that underlie cancer development, *e.g.,* cell dissociation, cell death and cell division.

Based on our previous biomarker studies, we were interested in studying MMP-10, a relatively understudied MMP in cancer biology. MMP-10 (also known as stromelysin 2) is generally limited to epithelial cells [3] and can target pro-MMP-1, -7, -8, -9, -13, collagen type III, IV, V, gelatin, elastin, fibronectin, proteoglycans and laminin [4], activities that have been shown to promote tumor cell invasion [5]. It has been demonstrated that MMP-10 expression is increased in several human tumors of epithelial origin, including gastric cancer [6,7], bladder cancer [8], esophageal cancer [9], skin cancer [10] and non-small cell lung cancer (NSCLC) [11]. These findings suggest that MMP-10 may play an important role in the development and progression of malignant tumors.

In this study, we monitored MMP-10 expression in cohorts of human tumor tissues, and investigated the mechanistic role of this MMP using a panel of *in vitro* and *in vivo* studies. We found that MMP-10 expression is positively correlated with an invasive phenotype in both human cervical and bladder cancers. Experimentally, we found that MMP-10 expression is tightly controlled and can be mediated by three-dimensional culture. We show that MMP-10 regulates migration/invasion capability, endothelial cell tube formation, and induces the expression of key angiogenic and metastatic factors (*e.g.,* MMP-9; hypoxia inducible factor-1 alpha, HIF-1α; chemokine (C-X-C motif) receptor 2, CXCR2; and plasminogen activator inhibitor-1, PAI-1). Furthermore, MMP-10 activity causes resistance to apoptosis via both the intrinsic and extrinsic apoptotic pathways. Lastly, we demonstrate that targeting MMP-10 in a human cervical cancer xenograft model with siRNA inhibited angiogenesis and induced apoptosis, resulting in a significant reduction in the growth of xenograft tumors. These results suggest that MMP-10 has distinct, multiple roles in tumor cell-matrix interactions that favor tumor progression.

## Methods

### Immunohistochemcal (IHC) staining of tissue microarrays

With IRB approval from MD Anderson Cancer Center Orlando, commercial tissue microarrays (TMA) (CR805 and BL2082, BL1002, US Biomax, Inc., Rockville, MD) constructed from clinical samples obtained from a cohort of 80 patients (70 cervical cancers; 67 adenocarcinoma and 3 squamous cell carcinoma and 10 benign cervical tissues) and from a cohort of 258 patients (188 bladder cancers and 70 benign bladder tissues) were examined by immunohistochemical staining. Clinical staging was recorded for cervical cancer using International Federation of Gynecology and Obstetrics (Stages 0-IV) and for bladder cancer using TNM staging (Stage I-IV). Protocol and antibody details are available in Additional file 1.

### Cells and reagents

Human cervical cancer cell line HeLa (adenocarcinoma from ATCC, Manassas, VA) and benign human bladder cell line, UROtsa (a generous gift from Dr. Donald Sens at the University of North Dakota School of Medicine, Grand Forks, ND) were available for analysis. HeLa cells were maintained in RPMI 1640 media and UROtsa cells were maintained in DMEM media as previously described [12]. Primary human umbilical vein endothelial cell (HUVEC, Cambrex) was cultured in EBM-2 basal media supplemented with EGM-2 MV Kit (Lonza) containing 2% FBS. HUVEC cells of passage 6 to 8 were used. To ensure optimal siRNA delivery in xenograft tumors, *in vivo*-jetPEI (Polyplus-transfection Inc. NYC, NY), a linear polyethylenimine, was used in conjunction with siRNA [13].

### Gene transfection for stable cell lines

A plasmid with a sequence verified human MMP-10 cDNA cloned within pCMV6-Entry vector and plasmid with vector alone (Origene Technologies) were transfected into HeLa cells using Fugene HD transfection reagent (Roche Diagnostics) to create HeLa-MMP-10^OE^ and HeLa^Empty,^ respectively. Stable transfectants were selected with 1,200 μg/ml of G418 (Life Technologies, Inc.) for 14 days and subcloned by limiting dilution in 96-well plates. Integration of the transfected gene into chromosome was confirmed by reverse transcriptase-PCR (*data not shown*). Stable cell lines were maintained in media containing 500 μg/ml of G418 for HeLa clones (HeLa-MMP-10^OE^ and HeLa^Empty^).

### Transfection of small interfering RNA (siRNA)

UROtsa cells were transfected for 6 hrs with synthesized MMP-10 siRNA (UROtsa-MMP-10^KD-1^ and UROtsa-MMP-10^KD-2^) or negative control siRNA (UROtsa-MMP-10^Scr^) (Life Technologies) using 6 well plates with a 100-pmol of siRNA and 5 μl of lipofectamine 2000 (Life Technology). Forty-eight hrs after transfection, transfected cells were replated for further experiments. MMP-10 siRNA sequences are listed in Additional file 1.

### Immunoblotting

Cell lysate and immunoblotting were performed using standard protocols as previously described [14]. Antibody details are available in Additional file 1.

### Quantitative reverse transcriptase-PCR

RNA was extracted from cells using RNeasy mini kit (Qiagen) as per manufacturer’s instructions. Conversion to cDNA was achieved through High Capacity cDNA Reverse Transcription kit (Life Technologies). Quantitative reverse transcriptase (RT)-PCR was carried out using ABI 7300 Real-Time PCR System (Life Technologies) in a 20 μl reaction volume containing 1 μl of the first-strand cDNA, 1 μM of gene-specific TaqMan primer and probe mix. Primer sets can be found in Additional file 1. Relative fold changes in mRNA levels were calculated after normalization to GAPDH using the comparative Ct method [15].

### Zymography

Thirty micrograms of total cell lysate from the parental cervical cancer cell line HeLa and parental urothelial cell line UROtsa as well as 30 μg of total cell lysate from the following clones: HeLa-MMP-10^OE^, HeLa^Empty^, UROtsa-MMP-10^KD1^&^2^ and UROtsa-MMP-10^Scr^, were electrophoresed on 10% SDS-polyacrylamide gels containing 1 mg/ml casein (Sigma, St. Louis, MO) under non-reducing conditions. After electrophoresis and SDS removal, MMP-10 renaturation was achieved by washing the gel for 1 hr in buffer containing 2.5% Triton-X 100, 50 mM tris pH 7.4, 5 mM CaCl_2_, and 1 μM ZnCl_2_. Gels were then incubated in a reaction buffer containing 50 mM Tris pH 7.4, 5 mM CaCl_2_, 1 μM ZnCl_2_ and 0.02% NaN_3_ pH 8.0 for 18 hrs at 37°C, followed by staining with Coomassie blue. MMP-10 activity was indicated by the degradation of casein. Gels were photographed using the KODAK Gel Logic 200 Imaging System with Carestream Molecular Imaging Software Standard Edition v5.0.7.24 (Carestream Health, Rochester, NY).

### Cell migration and invasion assays

Migration assays were performed in 6 well two-tier invasion chambers (Collaborative Biomedical Products, Bedford, MA, USA), using a protocol similar to that used successfully by our group [15]. Polycarbonate membranes were coated with 4 mg/ml growth factor reduced Matrigel (BD Biosciences, San Jose, CA) as described for invasion assays, control inserts (migration only) contained no coating. Two separate experimental designs were tested. First, HeLa-MMP-10^OE^, HeLa^Empty^ and UROtsa-MMP-10^KD1^&^2^, UROtsa-MMP-10^Scr^ cells were added to each insert at a density of 10^5^ cells/ml/well in RPMI media. The lower chamber contained RPMI media with 10% FBS as chemoattractant. After incubation in a humidified incubator in 5% CO_2_ at 37°C for 24 hrs, the cells on the top of the polycarbonate membrane were removed. The cells attached to the bottom of the membrane stained for 1 hr with cell viability indicator Calcein AM Fluorscent Dye (BD Biosciences, Franklin Lakes, NJ) and quantified using the FLUOstar OPTIMA at 495 mm excitation and 515 nm emission (BMG LABTECH Inc., Cary, NC).

In additional, HUVEC cells were added to each insert at a density of 10^5^ cells/ml/well in RPMI media. The lower chamber contained conditioned media from HeLa-MMP-10^OE^, HeLa^Empty^, UROtsa-MMP-10^KD1^&^2^ and UROtsa-MMP-10^Scr^. After 24 hrs, the HUVEC cells on the top of the polycarbonate membrane were removed, while the HUVEC cells attached to the bottom of the membrane were stained for 1 hr with cell viability indicator Calcein AM Fluorscent Dye and quantified using the FLUOstar OPTIMA. For the migration and invasion assays, at least three independent experiments consisting of each condition tested in triplicate wells was used to calculate mean ± SD values.

### Endothelial cell tube formation assays

Matrigel (BD Biosciences) was added to 96 well plates (40 μl per well) and allowed to solidify for 30 min at 37°C as previously described [16]. HUVEC cells were seeded on top of Matrigel in triplicates at a density of 10^4^ cells per well in conditioned media from cultured HeLa-MMP-10^OE^, HeLa^Empty^, UROtsa-MMP-10^KD1^&^2^ and UROtsa-MMP-10^Scr^ and allowed to incubate for 6 hrs. Next, images of capillary tube formation were captured using Leica DMIL inverted microscope. The angiogenic activities were quantitatively evaluated by measuring the total tube length of capillary tube in at least four viewed fields per well. At least three independent experiments consisting of each condition tested in triplicate wells was used to calculate mean ± SD values.

### RT^2^ Profiler PCR arrays for angiogenesis and metastasis

Cellular RNA from HeLa^Empty^ and HeLa-MMP-10^OE^ was recovered and converted to cDNA as described above. RT^2^ Profiler ‘human angiogenesis’ PCR arrays, (Catalog # PAHS-024ZA; SABiosciences) and RT^2^ Profiler ‘human metastasis’ PCR arrays, (Catalog # PAHS-028ZA; SABiosciences) were analyzed in duplicate according to the manufacturer's instructions (http://www.sabiosciences.com/pcrarraydataanalysis.php) by quantitative reverse transcriptase (RT)-PCR carried out using ABI 7300 Real-Time PCR system (Life Technologies). The specificity of the SYBR Green assay was confirmed by melting curve analysis. Relative fold changes in mRNA levels were calculated after normalization to housekeeping control gene targets using the comparative Ct method.

### Caspase activity assay

HeLa-MMP-10^OE^ and HeLa^Empty^ cells were lysed in caspase assay buffer containing 50 mM HEPES (pH 7.5), 100 mM NaCl, 2 mM EDTA, 0.1% CHAPS, 10% sucrose and 5 mM DTT. Aliquots of 6 mg of crude cell lysate were incubated with caspase-3 substrate Ac-DEVD-AMC (Pharmingen, San Diego, CA) at 37°C for 30 min. The caspase-3 activity was quantified in a FLUOstar Optima Reader (BMG LABTECH, Ortenberg, Germany) with excitation at 380 nm and emission at 440 nm. At least three independent experiments consisting of each condition tested in triplicate wells was used to calculate mean ± SD values.

### Annexin V apoptosis assay

HeLa-MMP-10^OE^ and HeLa^Empty^ cells were assessed in a LIVE/DEAD Annexin V apoptotic assay (BD Biosciences, Franklin Lakes, NJ) by flow cytometry. Furthermore, resistance to apoptosis was also determined in these same cells by exposing the cells to 1 μM of staurosporine (an agent capable of inducing apoptosis) for 5 hrs. In brief, after 5 hrs of exposure to staurosporine, cells were trypsinized, washed with 1 X PBS, and stained with propidium iodide (PI) in tandem with an APC-conjugated annexinV antibody according to the manufacturer’s instructions (BD Biosciences, San Jose, CA) and quantitated via flow cytometry using the BD FACScaliber with Cell Quest Pro Software (BD Biosciences, San Jose, California) and FlowJo (TreeStar Inc., Ashland, OR). Viable cells were negative for both annexin V and PI, early apoptotic cells exhibit externalization of phosphatidylserine and were annexin V positive but have an intact plasma membrane and were PI negative, while dead and damaged cells which have subsequently lost their membrane integrity were positive for both annexin V and PI.

### Mitochondrial membrane potential analysis

Analysis of mitochondrial membrane potential was performed as previously reported by our group [14]. Briefly, for the analysis of mitochondrial membrane potential (DCm), HeLa-MMP-10^OE^ and HeLa^Empty^ cells were seeded at 5 x 10^5^ cells in 10 cm tissue culture dishes for 24 hrs. Cells were then treated with 1 μM of staurosporine for 0, 1, 5 and 18 hrs. Next, cells were trypsinized, washed with 1 x PBS, incubated with medium containing JC-1 dye (10 μg/ml) for 20 min at 37°C. Lastly, the cells were washed and resuspended in 1 ml PBS for fluorescent flow cytometry analysis using FACScan flow cytometer (Becton Dickinson) measuring at least 10^4^ gated cells. Mitochondrial depolarization is indicated by a decrease in orange/green fluorescence ratio. All mitochondrial membrane potential analyses were performed in triplicate.

### Xenograft tumorgenicity

Animal care was in compliance with the recommendations of *The Guide for Care and Use of Laboratory Animals* (National Research Council) and approved by our local IACUC at the University of Central Florida and MD Anderson Cancer Center Orlando. First, the subcutaneous tumorigenicity assay was performed in athymic BALB/c (nu/nu) mice, 6 to 8 wks old purchased from Harlan Laboratories (Indianapolis, IN) by inoculating 10^6^ HeLa cells as described previously [17,18]. After two weeks, mice were divided randomly into four treatment groups (control, human siRNA MMP-10; 10 μg of siRNA with 1.2 μl of *in vivo*-jetPEI at a N/P ratio of 6 according to the manufacturer’s protocol, mouse siRNA MMP-10; 10 μg of siRNAs with 1.2 μl of *in vivo*-jetPEI at a N/P ratio of 6 and combination of human and mouse siRNA MMP-10; 5 μg of human siRNA and 5 μg of mouse siRNA with 1.2 μl of *in vivo*-jetPEI N/P ratio of 6) and treatment was initiated. siRNA therapy was administered intratumorally twice weekly for five weeks. Control mice received 10 μg of non-target siRNA mixed with 1.2 μl of *in vivo*-jetPEI N/P ratio of 6 on the same schedule. At least 10 animals were in each group. At the termination of the experiment, mice were sacrificed and tumors resected for IHC analysis.

### Immunohistochemical (IHC) analysis of xenograft tumors

Immunohistochemistry was conducted as described in refs. 17 and 18. Microvessel density (MVD) index was calculated as previously described [19]. Details and antibodies listed in Additional file 1.

### Statistical analyses

All data are expressed as mean ± standard deviation (SD). Statistical analyses were conducted using GraphPad Prism 5.0 (GraphPad Software, Inc.). For most comparisons, a 2-tailed unpaired Student *t* test or Mann-Whitney *U* test was conducted. The comparison between MMP-10 expression in low-grade, high-grade, low stage and high stage cancer was calculated using Fisher’s exact test. Differences were considered statistically significant at *p* < 0.05.

## Results

### MMP-10 expression is upregulated in cancer tissues

**Figure 1:**
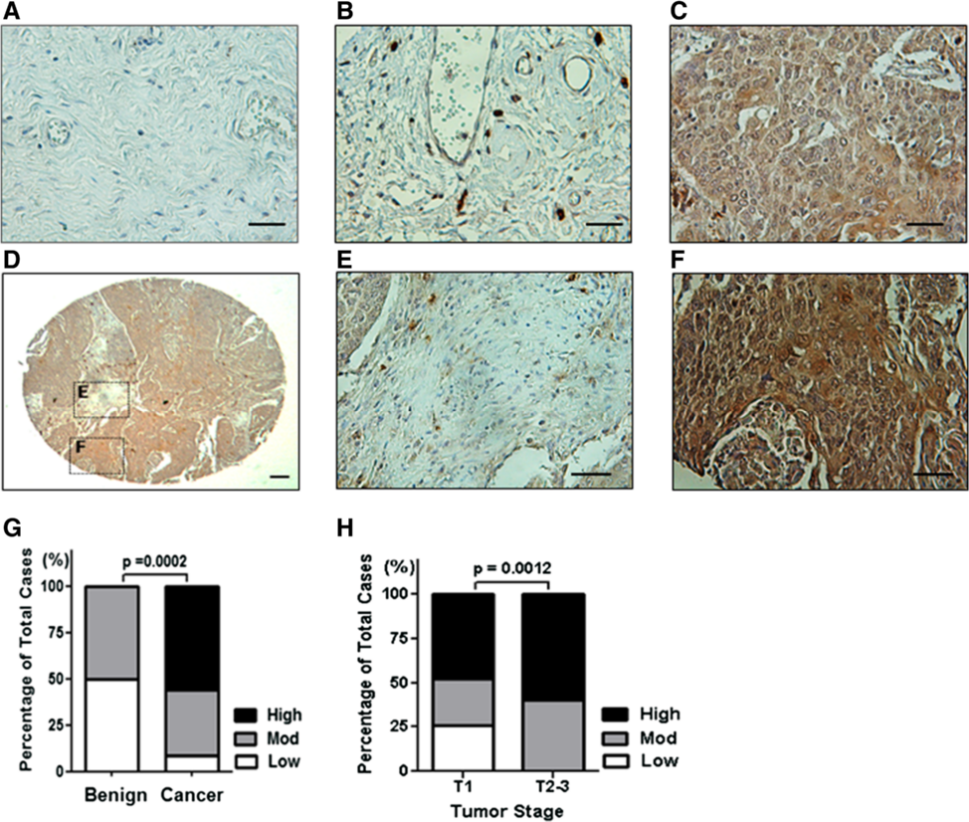
**MMP-10 expression is high in cervical cancer tissue.** Representative images of benign cervical tissue **(A)**, cervical tumor with weak **(B)**, and cervical tumor with strong **(C)** MMP-10 expression (brown). Representative image of a cervical tumor core on a tissue microarray **(D)**, MMP-10 expression in the tumor-associated stroma **(E)**, and in the tumor epithelia **(F)**. Scale bars, 100 μm. **G**, quantification of MMP-10 expression levels in benign cervical tissue (n = 10) *vs.* cervical tumor (n = 70)*.* Increased MMP-10 expression was noted in cervical tumors compared to benign cervical tissue. **H**, quantification of MMP-10 expression levels in low stage cervical tumors *vs.* high stage cervical tumors. Increased MMP-10 expression was noted in high stage cervical tumors.

We monitored MMP-10 protein expression in a panel of human cervical cancer tissues by immunohistochemical staining of a commercial TMA comprised of 10 benign specimens and 70 cancer tissues. Samples were semiquantitatively assessed by scoring the intensity of the staining and the proportion of MMP-10-positive cancer cells. Figure 1 shows representative negative images of benign samples (Figure 1A), and weakly (1+, Figure 1B) and strongly (3+, Figure 1C) positive staining in cervical tumor samples. We observed that MMP-10 was preferentially located in the epithelial component with scant, scattered staining in the stroma, as seen in individual biopsy cores (Figure 1D). Figure 1E provides a higher magnification of the stromal component, while Figure 1F illustrates the epithelial component. Analyses revealed low immunoreactivity for MMP-10 in the epithelial component of 50% of benign cervical tissue, and an absence of high immunoreactivity (0%) in benign cervical tissue. Conversely, low immunoreactivity for MMP-10 was present in the epithelial component of 8% of cancerous samples, and high immunoreactivity was present in 54% of cancerous samples, illustrating an increase in MMP-10 expression in cancerous tissues (Figure 1G, *p* = 0.0002). Notably, invasive cervical tumors showed significantly higher MMP-10 immunoreactivity compared to non-invasive tumors (Figure 1H, *p* = 0. 0012).

We have previously reported a significant increase in the expression of MMP-10 transcripts in shed urothelia from patients bearing bladder tumors relative to healthy controls [20], and we subsequently confirmed that increased soluble MMP-10 protein levels were indicative of bladder cancer in two large, independent studies [21,22]. In this study, we evaluated MMP-10 expression through immunohistochemical staining of a commercial bladder cancer TMA (70 benign samples and 188 cancer samples). Similar staining patterns to those found in the cervical cancers were noted in human bladder cancers (Additional file 2: Figure S1A-I). Specifically, 41 of the 71 samples from muscle invasive bladder cancer (MIBC) had moderate or high expression for MMP-10, whereas 30 samples had low or no expression. From the 68 non-muscle invasive bladder cancer (NMIBC) samples, 24 had moderate or high expression and 44 had low or no expression (*p* < 0.01 - Additional file 2: Figure S1I), indicating that MMP-10 expression increased in MIBC compared to NMIBC.

### MMP-10 expression stimulates migration, and invasion

**Figure 2:**
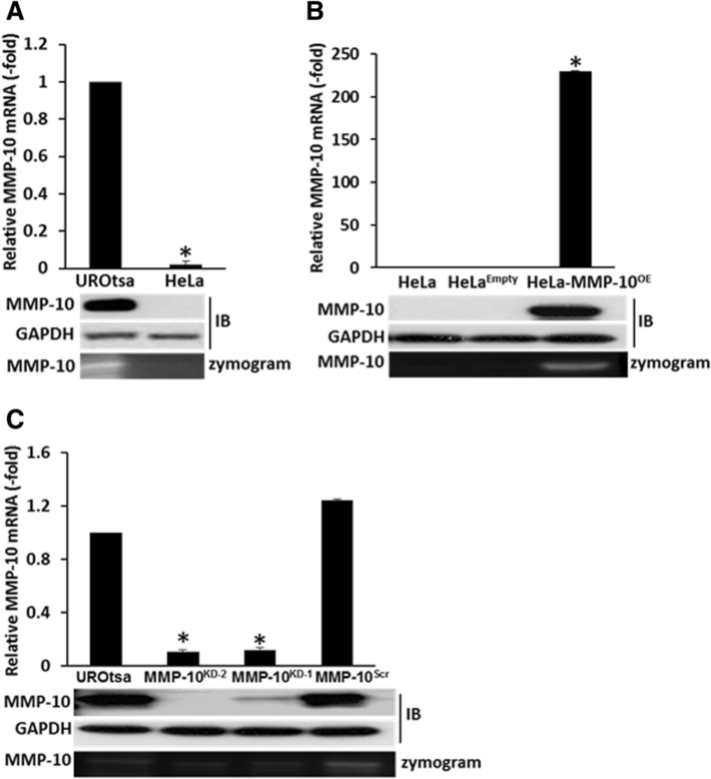
**MMP-10 expression in HeLa and UROtsa human cell lines. A**, MMP-10 expression was assessed by quantitative reverse transcriptase-PCR (qPCR), and immunoblot (IB) analyses in HeLa and UROtsa cells. MMP-10 activity was assessed by zymography, demonstrated by the degradation of casein. **B**, MMP-10 expression and activity was assessed by qPCR, immunoblot analyses and zymogram in stable HeLa clones; HeLa-MMP-10^OE^ overexpressing a functionally human MMP-10 in a pCMV6-Entry vector, and HeLa^Empty^ an empty-vector transfected control. **C**, MMP-10 assessed by qPCR, immunoblot and zymography in UROtsa clones; UROtsa-MMP-10^KD-1^, UROtsa-MMP-10^KD-2^ functional knockdown clones transfected with MMP-10 siRNA, and UROtsa-MMP-10^Scr^ transfected with a scrambled siRNA control.

We next investigated whether MMP-10 might contribute to key tumor cell processes including migration and invasion. Though other MMPs (*e.g.,* MMP-1, -2, -8, -9, -13) have been linked to degradation of laminin, vitronectin, fibronectin, enactin, collagen I-IV, MMP-10 has been identified as a stromelysin which can degrade various proteoglycan components of the ECM [23], thus facilitating tumor cell dissociation. Here, we utilized two human cell lines for experimental perturbation of MMP-10; the benign SV40 transformed urothelial line UROtsa and the cervical cancer cell line HeLa (Figure 2A). MMP-10 protein activity was demonstrated by zymography as evidenced by the degradation of casein (Figure 2A). Next, stable clones of HeLa cells that over-expressed MMP-10 (HeLa-MMP-10^OE^) and its corresponding control HeLa^Empty^ were created using plasmid DNA transfection (Figure 2B). Thus, we did not knockdown MMP-10 expression in human cervical cells. Stable clones of UROtsa cells with reduced MMP-10 expression (UROtsa-MMP-10^KD-1^, UROtsa-MMP-10^KD-2^) plus control line UROtsa-MMP-10^Scr^ were established using siRNA transfection (Figure 2C). Thus we did not overexpress MMP-10 in human bladder cells. MMP-10 expression in these cells was confirmed at both mRNA and protein levels, and activity was monitored by zymography. While the HeLa-MMP-10^OE^ clone had enhanced MMP-10 expression and activity, MMP-10 expression and activity were abrogated in both UROtsa-MMP-10^KD-1^ and UROtsa-MMP-10^KD-2^ cells relative to control UROtsa-MMP-10^Scr^ cells (Figure 2B & C).

**Figure 3:**
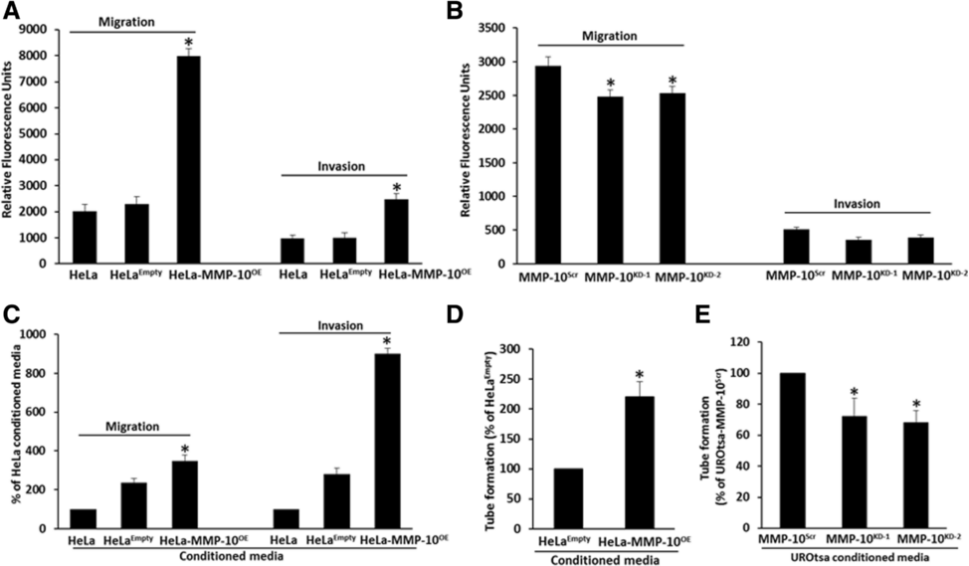
**MMP-10 promotes cellular migration and invasion. A**, HeLa cells (parental, HeLa^Empty^ and HeLa-MMP-10^OE^) and **B**, UROtsa cells (parental, UROtsa-MMP-10^KD-1^, UROtsa-MMP-10^KD-2^ and UROtsa-MMP-10^Scr^) were subjected to *in vitro* migration and invasion assays. Data are average values and SDs of three independent experiments conducted in triplicate. Overexpression of MMP-10 in HeLa-MMP-10^OE^ cells was noted to correspond with an increase in migratory and invasive potential, while silencing of MMP-10 in UROtsa-MMP-10^KD-1^ resulted in a reduced migratory potential. **C**, HUVEC cells were placed in the invasion chamber and then exposed to conditioned media in the lower chamber from HeLa cells (parental, HeLa-MMP-10^OE^ and HeLa^Empty^), and UROtsa cells (UROtsa-MMP-10^KD-1^, UROtsa-MMP-10^KD-2^ and UROtsa-MMP-10^Scr^) for *in vitro* migration and invasion assays. Data are average values and SDs of three independent experiments conducted in triplicate. Conditioned media from HeLa-MMP-10^OE^ cells that overexpressed MMP-10 was noted to induce migration and invasion of HUVEC cells. **D**, Capillary tube formation of HUVECs cultured in conditioned media from HeLa-MMP-10^OE^ and HeLa^Empty^ cells were quantified as total tube length per field in micrometers and was noted to be enhanced when MMP-10 was overexpressed. Data are average values and SDs of three independent experiments conducted in triplicate. **E**, Capillary tube formation in HUVECs cultured in conditioned media from UROtsa-MMP-10^KD-1^, UROtsa-MMP-10^KD-2^ and UROtsa-MMP-10^Scr^ cells were quantified as total tube length per field in micrometers and was noted to be inhibited when MMP-10 was silenced. Data are average values and SDs of three independent experiments conducted in triplicate. *, *p* < 0.05.

In an *in vitro* migration and invasion assay, the migratory potential of HeLa-MMP-10^OE^ was significantly enhanced compared to HeLa^Empty^ (*p* = 0.012, Figure 3A). Similarly, the migratory potential of UROtsa-MMP-10^KD-1^ and UROtsa-MMP-10^KD-2^ cells was significantly reduced compared to UROtsa-MMP-10^Scr^ (*p* < 0.05, Figure 3B). The same phenomena were observed in the invasion assay, HeLa-MMP-10^OE^ cells having significant enhancement of invasion compared to HeLa^Empty^ cells (*p* = 0.002, Figure 3A). No significant difference in invasive potential was noted in UROtsa-MMP-10^KD-1^ and UROtsa-MMP-10^KD-2^ compared to UROtsa-MMP-10^Scr^ (Figure 3B).

### MMP-10 influences endothelial cell behavior

We also evaluated the ability of MMP-10 to influence the migration and invasion of human endothelial cells. HUVEC cells were tested for movement towards conditioned media from parental HeLa, HeLa^Empty^, and HeLa-MMP-10^OE^ cells in the assay described above. For HUVEC cells, the conditioned media associated with HeLa-MMP-10^OE^ caused an increase in migration (*p* = 0.013) and invasion (*p* = 0.002) compared to conditioned media from HeLa^Empty^ cells (Figure 3C). No significant difference in migratory or invasive potential was noted in HUVEC cells exposed to conditioned media from UROtsa-MMP-10^KD-1^ and UROtsa-MMP-10^KD-2^ compared to UROtsa-MMP-10^Scr^ or parental UROtsa cells (data not shown).

As a surrogate for potential effects on angiogenesis, we set out to investigate whether MMP-10 expression might influence endothelial tube formation. Conditioned media from the test cell lines was utilized in an *in vitro* tube formation assay. The total length of tube-like structures formed by HUVECs on growth factor-reduced Matrigel was significantly enhanced by treatment with conditioned media from HeLa-MMP-10^OE^ cells compared to HeLa^Empty^ cells (~220%, Figure 3D). Conversely, the total length of tube-like structures was significantly reduced when HUVECs were treated with conditioned media from UROtsa-MMP-10^KD-1^ and UROtsa-MMP-10^KD-2^ (~72% and ~68%, respectively; Figure 3E) compared to UROtsa-MMP-10^Scr^.

**Figure 4:**
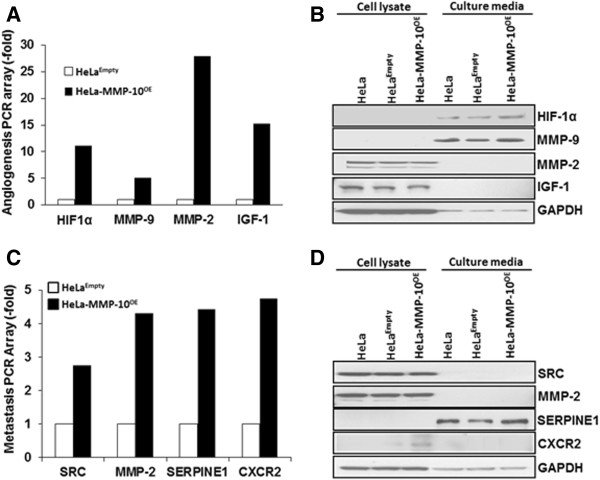
**MMP-10 regulates the expression of key angiogenic and metastatic factors. A**, Angiogenesis RT^2^ Profiler PCR Arrays evaluated by quantitative RT-PCR assessed the expression of 84 gene transcripts. Gene expression levels were normalized to the combination of five housekeeping genes (β-actin, B2M, GAPDH, HPRT and RPLP). Changes in expression of HIF-1α, MMP-9, MMP-2, IGF-1 transcripts (see other changes listed in Additional file 3: Table S1) were noted in HeLa-MMP-10^OE^ cells relative to HeLa^Empty^ cells. **B**, Western blot analysis confirmed the differential expression of HIF-1α and MMP-9 at the protein level. GAPDH served as a loading control. **C**, Metastasis RT^2^ Profiler PCR Arrays evaluated by quantitative RT-PCR assessed 84 gene transcripts. Gene expression levels were normalized to the combination of five housekeeping genes (β-actin, B2M, GAPDH, HPRT and RPLP). Changes in expression of Src, MMP-2, PAI-1, CXCR2 (also see Additional file 4: Table S2) were noted in HeLa-MMP-10^OE^ cells relative to HeLa^Empty^ cells. **D**, Western blot analysis confirmed differential protein expression of PAI-1 and CXCR2. GAPDH served as a loading control.

To follow up on these observed effects we profiled the expression of key angiogenic and metastatic factors in the manipulated cell lines using commercial PCR arrays. Significant differential expression was observed for many gene transcripts in HeLa-MMP-10^OE^ cells relative to the HeLa^Empty^ control line. Among the MMP-10 induced genes implicated in angiogenesis (Figure 4A and Additional file 3: Table S1), differential expression of HIF-1α and MMP-9 was confirmed by Western blot analysis (Figure 4B). Among a panel of genes identified from the metastasis PCR array (Figure 4C and Additional file 4: Table S2), differential expression of CXCR2 and PAI-1 was confirmed by Western blot (Figure 4D). Thus, the expression of MMP-10 was associated with the upregulation of key angiogenic and metastatic proteins in HeLa cells, factors which could facilitate tumor growth and progression.

### MMP-10 influences apoptosis

**Figure 5:**
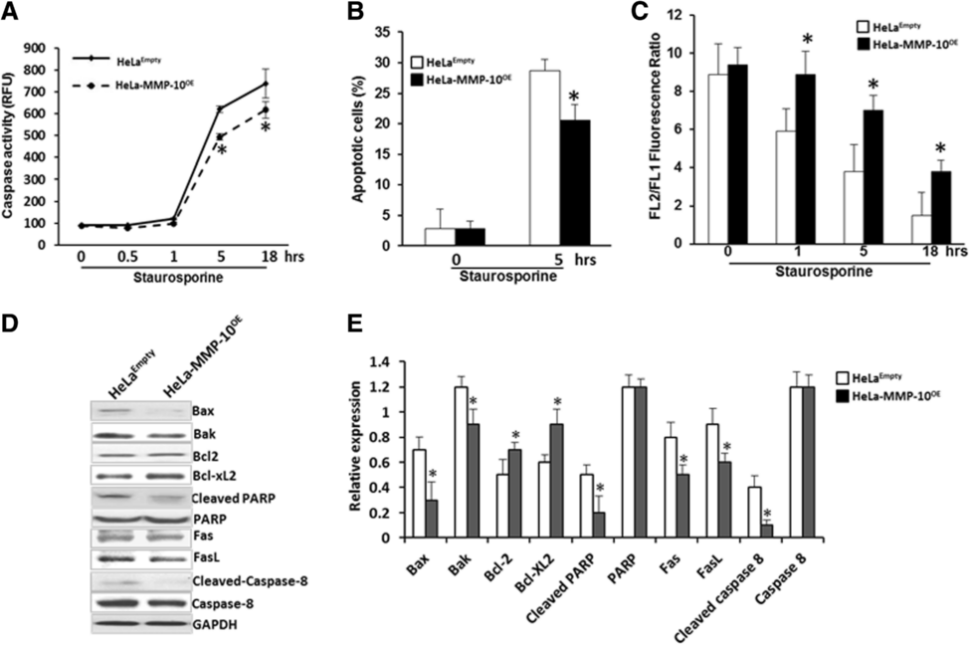
**Overexpression of MMP-10 results in inhibition of apoptosis. A**, Caspase activity assay was performed with HeLa-MMP-10^OE^ and HeLa^Empty^ cells after exposing the cells to the apoptotic-inducing agent, staurosporine at 1 μM. At least three independent experiments performed in triplicate were used to calculate mean ± SD values. *, *p* < 0.05. The apoptotic effects of staurosporine were abrogated by MMP-10 overexpression. **B**, Annexin V apoptotic assays were also performed with HeLa-MMP-10^OE^ and HeLa^Empty^ cells as described above. Assays confirmed that high expression of MMP-10 was associated with resistance to induced apoptosis. **C**, Changes in mitochondrial membrane potential (DCm) in HeLa-MMP-10^OE^ and control HeLa^Empty^ cells were assessed by flow cytometric analysis. At least three independent experiments performed in triplicate wells were used to calculate mean ± SD values. *, *p* < 0.05. High expression of MMP-10 was associated with a significant increase in DCm compared to control. **D**, Western blot analysis was performed on the above cell lines confirming MMP-10 overexpression was associated with the expression of apoptosis factors. For example, the expression of key intrinsic apoptotic pathway factors (Bcl-xl, Bax and Bak), and factors in the extrinsic apoptotic pathway (FasL and cleaved caspase-8) were altered when MMP-10 was overexpressed. GAPDH served as controls. **E**, The expression of the proteins from the Western blot from panel D was quantitated and plotted to illustrate relative expression of the apoptotic associated proteins.

Previous investigators have reported a link between MMP-9 and MMP-10 and an ability to induce p53-mediated apoptosis [24]. Here, we set out to determine if MMP-10 could regulate apoptosis within HeLa cells that have wild-type p53. Using a caspase activity assay, we investigated whether overexpression of MMP-10 in HeLa cells resulted in a reduction in apoptosis in the presence and absence of the apoptosis-inducing agent, staurosporine (1 μM) (Additional file 5: Figure S2). HeLa-MMP-10^OE^ cells were noted to have a 21% reduction in caspase activity over HeLa^Empty^ cells (*p* = 0.025, Figure 5A). Similarly in an Annexin V apoptosis assay, the percentage of cell undergoing apoptosis was reduced by 28% in HeLa-MMP-10^OE^ cells compared to HeLa^Empty^ cells (*p* = 0.029) (Figure 5B). Analysis of mitochondrial membrane potential (DCm) revealed a significantly higher DCm, as noted by the increase in FL2/FL1 fluorescence ratio, in HeLa-MMP-10^OE^ relative to HeLa^Empty^ cells (Figure 5C). HeLa-MMP-10^OE^ cells proved to possess a higher DCm at all time points post-staurosporine treatment, confirming a role for MMP-10 in resistance to staurosporine-induced apoptosis. Furthermore, we assessed the expression of a panel of apoptotic factors implicated in either the intrinsic or extrinsic apoptosis pathways (Figure 5D). Western blot data revealed that when MMP-10 was overexpressed, members of the intrinsic pathway; Bax and Bak were reduced, while Bcl-xl was increased. Monitoring members of the extrinsic apoptosis pathway revealed that MMP-10 overexpression was associated with a reduction in FasL and cleaved caspase-8. Thus, this is the first report demonstrating MMP-10 activity confers HeLa cell resistance to apoptosis through multiple factors related to the intrinsic and extrinsic apoptosis pathway.

### MMP-10 siRNA therapy inhibits xenograft tumor growth

**Figure 6:**
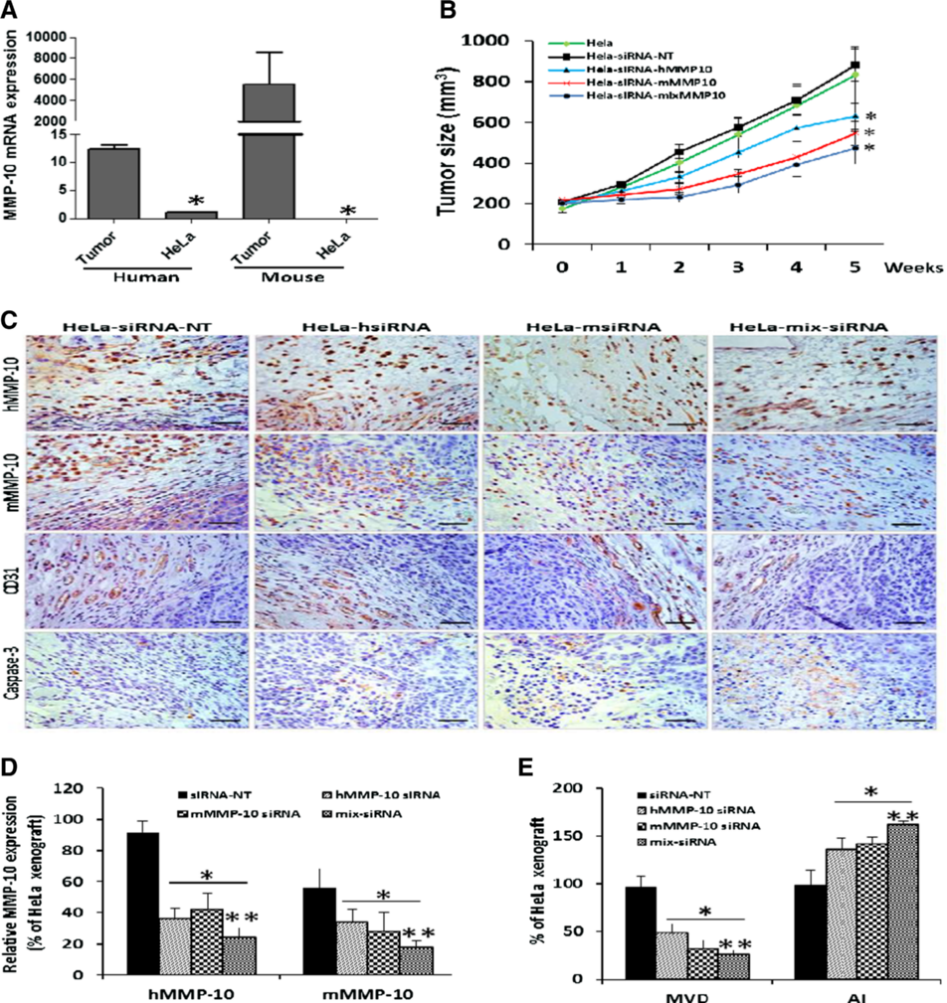
**MMP-10 regulates HeLa xenograft tumorigenicity. A**, Human and mouse MMP-10 expression in subcutaneous HeLa tumors and in parental HeLa cells grown in culture was assessed by qPCR. Gene expression level was normalized to GAPDH. Cultured parental HeLa cells expressed minimal MMP-10, but when grown in a subcutaneous mouse model, increases in both human and mouse MMP-10 were noted. **B**, Tumor growth was established by subcutaneous injection of parental HeLa cells into athymic nude mice (nu/nu). PBS (negative control), scrambled siRNA (negative control), human MMP-10 siRNA and murine MMP-10 siRNA reagents were injected intratumorally twice weekly as described in Materials and Methods. Tumor size was plotted as mean ± SD from the four treatment groups (n = 10/group). Treatment with siRNA MMP-10 human alone, siRNA MMP-10 mouse alone and the combination of human and mouse siRNA MMP-10 were associated with a significant reduction in tumor burden. *, *p* < 0.05. **C**, Human MMP-10 and mouse MMP-10 expression in excised tumors are shown (images 400 ×, Bar = 50 μm). A reduction in MMP-10 and CD31 expression along with an induction of cleaved caspase-3 was noted in tumors treated with human MMP-10 siRNA alone, mouse MMP-10 siRNA alone and the combination of human and mouse siRNAs. **D**, MMP-10 expression was quantified based on MMP-10 staining. **E**, Microvessel density (MVD) was quantified based on CD31 staining. Apoptotic index (AI) was quantified based on cleaved caspase-3 staining. Data are presented as mean ± SD, *, *p* < 0.05 compared to negative control.

HeLa cells form robust subcutaneous xenografts in athymic mice. Following our *in vitro* findings, we hypothesized that perturbation of MMP-10 *in vivo* could influence the tumorigenicity of HeLa cell xenografts. Though parental HeLa cells were found to express very low levels of MMP-10 in monolayer cultures, in preliminary xenograft tests, we noticed that MMP-10 levels significantly increased as three-dimensional HeLa xenograft tumors were established. Moreover, MMP-10 from both human and mouse sources were evident in xenograft tissue, with mouse MMP-10 expression being at a high level (Figure 6A). Thus, when we set out to inhibit MMP-10 in the xenograft experiments, we targeted both human and mouse MMP-10 using distinct siRNA reagents.

Mice in each test group were injected intratumorally twice weekly with appropriate siRNA. At the end of the 5-week study, untreated HeLa xenografts, and xenografts treated with a scrambled mouse and human control siRNA reagent mix, reached an average volume of 833 mm^3^ and 882 mm^3^, respectively (Figure 6B). HeLa xenografts treated with human MMP-10 siRNA reached an average volume of 629 mm^3^ (29% reduction compared to control siRNA, *p* < 0.001), while HeLa xenografts treated with mouse MMP-10 siRNA established tumors of 545 mm^3^ volume (38% reduction compared to control siRNA, *p* < 0.001). HeLa xenografts treated with a combined human and mouse MMP-10 siRNA mix established tumors of only 474 mm^3^, a 46% reduction compared to control (*p* < 0.001), however, this was not significantly different from either human siRNA MMP-10 or mouse siRNA MMP-10 treatment alone (Figure 6B). A clear reduction in MMP-10 expression was identified in xenograft tumors treated with either human or mouse siRNA MMP-10 (Figure 6C & D). Interestingly, when tumors were treated with either human or mouse MMP-10 siRNA alone, a reduction in the expression of the other species MMP-10 was also noted (Figure 6D). Cross reactivity between the species-specific siRNAs was not noted *in vitro* (data not shown), thus, we speculate that there is some positive feedback link between mouse stromal MMP-10 and HeLa tumor cell MMP-10 production.

Expression levels of the vasculature marker CD31 were monitored in xenograft sections to establish a microvessel density (MVD) index. Analyses revealed a severe reduction of CD31 positivity in HeLa xenograft tumors treated with either human or mouse MMP-10 siRNA. MVD calculated in xenografts treated with the human MMP-10 siRNA, the mouse MMP-10 siRNA, and the combination of human and mouse MMP-10 siRNA groups, revealed a reduction of 50%, 66% and 73%, respectively, relative to control siRNA treatment (Figure 6E). Lastly, the expression level of caspase-3 was evaluated to estimate an apoptotic index (AI). AI of xenografts treated with the human MMP-10 siRNA, the mouse MMP-10 siRNA, and the combination of human and mouse MMP-10 siRNA confirmed an increase of 38%, 45% and 87%, respectively, compared to control siRNA treatment (Figure 6E). However, the combination of human and mouse MMP-10 siRNA did not result in significantly altered MVD or AI relative to those treated with human or mouse MMP-10 siRNA alone. The *in vivo* observations corroborate the *in vitro* findings and confirm a role for MMP-10 in the provision of a pro-tumor growth environment through an influence on angiogenesis and apoptosis.

## Discussion

Our findings reveal that MMP-10 expression can induce the expression of key molecules implicated in angiogenesis, metastasis and apoptosis, major mechanisms involved in the establishment and progression of malignant tumors. Though MMP-10 is well characterized biochemically, compared to other MMPs, little is known about MMP-10 in human cancers. Here, we are the first to report the relative increased expression of MMP-10 in human cervical cancer compared to benign tissue, and that expression correlates with the invasive phenotype of malignant cervical tumors. Given that we have recently demonstrated that MMP-10 expression at the RNA [20] and protein level [21,22] is increased in urine samples obtained from patients with bladder cancer, so there appears be a trend of association between malignancy and MMP-10. Within this study, analysis of 188 tumor specimens confirmed that MMP-10 protein expression was increased in human bladder tumors, and, as in cervical cancer study, a correlation between more aggressive bladder cancers and MMP-10 expression was noted (Additional file 2: Figure S1). Only one small study has previously reported the expression of MMP-10 in human bladder tumors. In that study, Seargent et al., demonstrated that MMP-10 was predominantly present in the epithelial component of tumor tissue, and that bladder cancer had significantly greater levels of MMP-10 than benign bladder specimens. However, the investigators could not demonstrate a difference in MMP-10 expression between low stage and high stage disease, possibly due to the small (n = 60) sample size [7]. Limited data are available for MMP-10 expression in cervical tissue. However in a small study, Vazquez-Ortiz et al. reported that overexpression of MMP-10 was observed to be consistently overexpressed in invasive cervical cancer biopsies associated by cDNA array. Validation on an independent, large cohort was not performed [25]. We must stress that HPV status was not reported on the commercial TMA utilized in our study. It would be interesting to investigate whether MMP-10 expression is correlated with disease prognosis in cervical and bladder cancers, as it has been in esophageal carcinoma [26], current studies to address this are underway.

MMP-10 (also known as stromelysin 2) is generally limited to epithelial cells and its regulation is tightly controlled [27]. For example, MMP-10 is not present in intact normal skin, but is expressed during cutaneous injury and repair where it is localized to the migrating keratinocytes at the wound edge, alluding to its role as a pro-invasion molecule [5]. In the HeLa cervical cancer cell line, MMP-10 was not expressed in culture. However, when HeLa cells were grown in a 3D matrix (data not shown), or as a xenograft tumor (Figure 6A), MMP-10 expression was induced. This is similar to the report by Mishra et al., who demonstrated that A549 human lung cancer cells grown in an *ex vivo* 3D model expressed significantly more MMP-10 than when grown as a monolayer culture [28]. Though benign (as defined by inability to form xenograft tumors), the UROtsa cell line has been transformed by SV40 [29], and thus has a non-functional p53. This could account for the high level of MMP-10 expression, supporting a role for p53 in MMP-10 expression, originally noted by Meyer et al. [24].

A considerable amount of information has accumulated to suggest that the role of MMPs in cancer is far more complicated than initially presumed. One such role in cancer is related to apoptosis. MMP activity may have proapoptotic or antiapoptotic effects depending on the local cellular production and availability of proteolytically released and activated death-inducing and/or growth and survival factors [30]. For example, proapoptotic effects can be due to degradation of ECM proteins that serve as ligands for specific classes of integrins. In the absence of the ligand, integrins can no longer trigger signals necessary for epithelial and endothelial survival thus resulting in apoptosis. Alternatively, ECM degradation can result in the release of IGF-1, which could directly promote tumor cell resistance to proapototic signals. In our study, HeLa cells (p53 wild-type) were found to be more resistant to apoptosis when MMP-10 was expressed. This resistance to apoptosis was associated with changes in the expression of key molecules in both the intrinsic (*e.g.,* reduction in Bax, Bak and cleaved caspase 8 and an increase in Bcl-xl) and extrinsic apoptotic pathway *(e.g.,* reduction in FasL and cleaved caspase 8).

An interesting co-expression relationship that we were able to confirm was that between MMP-10 and PAI-1. PAI-1 is an important endogenous inhibitor of urokinase-type plasminogen activator. Specifically, PAI-1 normally functions as part of the plasminogen activation (PA) system, which includes the serine protease uPA (urokinase-type plasminogen activator), its receptor uPA-R, tPA (tissue plasminogen activator) and inhibitors PAI-1 and PAI-2 [31]. PAI-1 expression may be regulated by many intrinsic factors (*e.g.,* cytokines and growth factors) and extrinsic factors (*e.g.,* cellular stress) [32]. Although the canonical function of PAI-1 has been known as an inhibitor of uPA to maintain clot formation [33] it is now regarded as a pleiotropic factor exerting diverse cellular effects, many related to tumorigenesis; cellular proliferation, migration, invasion, adhesion and angiogenesis. The deregulation of PAI-1 has been correlated to several tumor types (*e.g.,* breast, colorectal, lung and bladder) and has been associated as a poor prognostic factor [34-40]. Previous studies have reported a positive correlation between MMP-10 and PAI-1 [41-43]. Similar, we report an increase of both MMP-10 and PAI-1 in voided urine samples from subjects with bladder cancer [21,22]. Interesting, Wilkins et al. reported that the addition of PAI-1 blocks collagen degradation as well as the conversion of MMP-10 to its catalytically active form. Thus there exist MMP-10/PAI-1/plasmin dependent proteolytic axis that enhances ECM degradation and facilitates angiogenesis and invasion. Further characterization of this axis may have far reaching implications for therapeutic targeting of human malignancies.

## Conclusions

In a series of *in vitro* and *in vivo* experiments, MMP-10 activity was shown to be pivotal in the tumor growth in a mouse model of cancer, and the expression of this MMP is associated with the upregulation of key molecules related to angiogenesis, metastasis, and apoptosis, creating a milieu favorable to the survival and expansion of malignant lesions. Furthermore, demonstration of a correlation between MMP-10 expression and invasive cervical and bladder cancers supports a role for MMP-10 in human tumor progression. These data suggest that MMP-10 may have value as a novel biomarker and/or provide a molecular target for therapeutic intervention.

## Competing interest

S. Goodison and C.J. Rosser are officers in Nonagen Bioscience Corporation. All other authors declare that they have no competing interests.

## Authors’ contributions

GZ Perform in vitro and in vivo experiments and analysis. MM Perform in vitro and in vivo experiments and analysis. AL Pathologic review of human and animal tissues. Steve Goodison Study concept and design, drafting of manuscript. CJR Study concept and design, drafting of manuscript, administrative support and funding. All authors read and approved the final manuscript.

## Pre-publication history

The pre-publication history for this paper can be accessed here:

http://www.biomedcentral.com/1471-2407/14/310/prepub

## Supplementary Material

Additional file 1 Immunohistochemcal evaluation of tissue microarrays. Click here for file

Additional file 2: Figure S1 MMP-10 expression pattern in human bladder cancer. Representative images of normal urothelium (A) and of bladder cancers with absent (B), weak (C), and strong (D) MMP-10 expression (brown) are shown. Representative images of a bladder tissue specimen core on a tissue microarray (E), MMP-10 expression in the tumor stroma (F), and MMP-10 expression in the tumor epithelia (G). Scale bars, 100 μm. H, quantification of MMP-10 expression levels in benign bladder tissue (n = 70) *vs.* bladder tumor (n = 188). I, quantification of MMP-10 expression levels in non-muscle invasive bladder cancer (NMIBC) *vs*. muscle invasive bladder cancer (MIBC). Increased MMP-10 expression was noted in bladder tumors compared to benign bladder tissue and the expression levels were noted to be increased in MIBC compared to NMIBC. Click here for file

Additional file 3: Table S1 RT^2^ Profiler PCR Array for Angiogenesis (HeLa-MMP-10^OE^/HeLa^Empty^). Click here for file

Additional file 4: Table S2 RT2 Profiler PCR Array for Metastasis (HeLa-MMP-10OE/HeLaEmpty). Click here for file

Additional file 5: Figure S2 Apoptotic activity in HeLa cells overexpressing MMP-10. A) Caspase activity assay in HeLa^empty^ and HeLa-MMP-10^OE^. B) Percentage of cells undergoing apoptosis in HeLa^empty^ and HeLa-MMP-10^OE^. C) Percentage of HeLa^empty^ and HeLa-MMP-10^OE^ cells undergoing apoptosis after exposure to external stressors. Click here for file

## References

[B1] Hojilla CV Mohammed FF Khokha R Matrix metalloproteinases and their tissue inhibitors direct cell fate during cancer development Br J Canc 2003 89 1817 1821 10.1038/sj.bjc.6601327 PMC2394437 14612884

[B2] Egeblad M Werb Z New functions for the matrix metalloproteinases in cancer progression Nat Rev Cancer 2002 2 161 174 10.1038/nrc745 11990853

[B3] Madlener M Mauch C Conca W Brauchle M Parks WC Werner S Regulation of the expression of stromelysin-2 by growth factors in keratinocytes: implications for normal and impaired wound healing Biochem J 1996 320 659 664 8973581 10.1042/bj3200659 PMC1217980

[B4] Chakraborti S Mandal M Das S Mandal A Chakraborti T Regulation of matrix metalloproteinases: an overview Mol Cell Biochem 2003 253 269 285 10.1023/A:1026028303196 14619979

[B5] Krampert M Bloch W Sasaki T Bugnon P Rülicke T Wolf E Bloch W Sasaki T Bugnon P Rülicke T Wolf E Activities of the matrix metalloproteinase stromelysin-2 (MMP-10) in matrix degradation and keratinocyte organization in wounded skin Mol Biol Cell 2004 15 5242 5254 10.1091/mbc.E04-02-0109 15371548 PMC532007

[B6] Aung PP Oue N Mitani Y Nakayama H Yoshida K Noguchi T Bosserhoff AK Yasui W Systematic search for gastric cancer-specific genes based on SAGE data: melanoma inhibitory activity and matrix metalloproteinase-10 are novel prognostic factors in patients with gastric cancer Oncogene 2006 25 2546 2557 10.1038/sj.onc.1209279 16331256

[B7] Eck M Schmausser B Scheller K Brändlein S Müller-Hermelink HK Pleiotropic effects of CXC chemokines in gastric carcinoma: differences in CXCL8 and CXCL1 expression between diffuse and intestinal types of gastric carcinoma Clin Exp Immunol 2003 134 508 515 10.1111/j.1365-2249.2003.02305.x 14632759 PMC1808898

[B8] Seargent JM Loadman PM Martin SW Naylor B Bibby MC Gill JH Expression of matrix metalloproteinase-10 in human bladder transitional cell carcinoma Urology 2005 65 815 820 10.1016/j.urology.2004.11.016 15833553

[B9] Mathew R Khanna R Kumar R Mathur M Shukla NK Ralhan R Stromelysin-2 overexpression in human esophageal squamous cell carcinoma: potential clinical implications Canc Detect Prev 2002 26 222 228 10.1016/S0361-090X(02)00035-1 12269770

[B10] Kerkelä E Ala-aho R Lohi J Grénman R M-Kähäri V Saarialho-Kere U Differential patterns of stromelysin-2 (MMP-10) and MT1-MMP (MMP-14) expression in epithelial skin cancers Br J Canc 2001 84 659 669 10.1054/bjoc.2000.1634 PMC2363801 11237387

[B11] Zhang X Zhu S Luo G Zheng L Wei J Zhu J Hu S Expression of MMP-10 in lung cancer Anticancer Res 2007 27 2791 2795 17695449

[B12] Miyake M Goodison S Giacoia EG Rizwani W Ross S Rosser CJ Influencing factors on the NMP-22 urine assay: an experimental model BMC Urol 2012 12 23 doi:10.1186/1471-2490-12-23 10.1186/1471-2490-12-23 22928931 PMC3480828

[B13] Ito T Shimada Y Kan T David S Cheng Y Mori Y Agarwal R Paun B Jin Z Olaru A Hamilton JP Yang J Abraham JM Meltzer SJ Sato F Pituitary tumor-transforming 1 increases cell motility and promotes lymph node metastasis in esophageal squamous cell carcinoma Canc Res 2008 68 3214 3218 10.1158/0008-5472.CAN-07-3043 PMC2988648 18451147

[B14] Cao W Yacoub S Shiverick KT Namiki K Sakai Y Porvasnik S Urbanek C Rosser CJ Dichloroacetate (DCA) sensitizes both wild-type and over expressing Bcl-2 prostate cancer cells in vitro to radiation Prostate 2008 68 1223 1231 10.1002/pros.20788 18465755

[B15] Pfaffl MW A new mathematical model for relative quantification in real-time RT-PCR Nucleic Acids Res 2001 29 e45 10.1093/nar/29.9.e45 11328886 PMC55695

[B16] Anai S Sakamoto N Sakai Y Tanaka M Porvasnik S Urbanek C Cao W Goodison S Rosser CJ Dual targeting of Bcl-2 and VEGF: a potential strategy to improve therapy for prostate cancer Urol Oncol 2011 29 421 429 10.1016/j.urolonc.2009.04.009 19576799

[B17] Namiki K Goodison S Porvasnik S Allan RW Iczkowski KA Urbanek C Reyes L Sakamoto N Rosser CJ Persistent exposure to Mycoplasma induces malignant transformation of human prostate cells PLoS One 2009 4 e6872 doi:10.1371/journal.pone.0006872 10.1371/journal.pone.0006872 19721714 PMC2730529

[B18] Anai S Goodison S Shiverick K Hirao Y Brown BD Rosser CJ Knock-down of Bcl-2 by antisense oligodeoxynucleotides induces radiosensitization and inhibition of angiogenesis in human PC-3 prostate tumor xenografts Mol Canc Ther 2007 6 101 111 10.1158/1535-7163.MCT-06-0367 17237270

[B19] Weidner N Folkman J Pozza F Bevilacqua P Allred EN Moore DH Meli S Gasparini G Tumor angiogenesis: a new significant and independent prognostic indicator in early stage breast carcinoma J Natl Canc Inst 1992 84 1875 1887 10.1093/jnci/84.24.1875 1281237

[B20] Urquidi V Goodison S Cai Y Sun Y Rosser CJ A candidate molecular biomarker panel for the detection of bladder cancer Canc Epidemiol Biomarkers Prev 2012 21 2149 2158 10.1158/1055-9965.EPI-12-0428 PMC3537330 23097579

[B21] Goodison S Chang M Dai Y Urquidi V Rosser CJ A multi-analyte assay for the non-invasive detection of bladder cancer PLoS One 2012 7 10 e47469 10.1371/journal.pone.0047469 23094052 PMC3477150

[B22] Rosser CJ Ross S Chang M Dai Y Mengual L Zhang G Kim J Urquidi V Alcaraz A Goodison S Rosser CJ Multiplex protein signature for the detection of bladder cancer in voided urine samples J Urol 2013 5347 4581 4583 10.1016/j.juro.2013.06.011 PMC4013793 23764080

[B23] Zucker S Cao J Chen WT Critical appraisal of the use of matrix metalloproteinase inhibitors in cancer treatment Oncogene 2000 19 6642 6650 10.1038/sj.onc.1204097 11426650

[B24] Meyer E Vollmer JY Bovey R Stamenkovic I Matrix metalloproteinases 9 and 10 inhibit protein kinase C-potentiated, p53-mediated apoptosis Canc Res 2005 65 4261 4272 10.1158/0008-5472.CAN-04-2908 15899818

[B25] Vázquez-Ortíz G Ciudad CJ Piña P Vazquez K Hidalgo A Alatorre B Garcia JA Salamanca F Peralta-Rodriguez R Rangel A Salcedo M Gene identification by cDNA arrays in HPV-positive cervical cancer Arch Med Res 2005 36 448 458 10.1016/j.arcmed.2005.04.016 16099320

[B26] Liu H Qin YR Bi J Guo A Fu L Guan XY Overexpression of matrix metalloproteinase 10 is associated with poor survival in patients with early stage of esophageal squamous cell carcinoma Dis Esophagus 2012 25 656 663 10.1111/j.1442-2050.2011.01284.x 22121946

[B27] Sternlicht MD Werb Z How matrix metalloproteinases regulate cell behavior Annu Rev Cell Dev Biol 2001 17 463 516 10.1146/annurev.cellbio.17.1.463 11687497 PMC2792593

[B28] Mishra DK Sakamoto JH Thrall MJ Baird BN Blackmon SH Ferrari M Kurie JM Kim MP Human lung cancer cells grown in an ex vivo 3D lung model produce matrix metalloproteinases not produced in 2D culture PLoS One 2012 7 e45308 doi:10.1371/journal.pone.0045308 10.1371/journal.pone.0045308 23028922 PMC3444466

[B29] Rossi MR Masters JR Park S Todd JH Garrett SH Sens MA Somji S Nath J Sens DA The immortalized UROtsa cell line as a potential cell culture model of human urothelium Environ Health Perspect 2001 109 801 808 10.1289/ehp.01109801 11564615 PMC1240407

[B30] McCawley LJ Matrisian LM Matrix metalloproteinases: they're not just for matrix anymore! Curr Opin Cell Biol 2001 13 534 540 10.1016/S0955-0674(00)00248-9 11544020

[B31] Rijken DC Plasminogen activators and plasminogen activator inhibitors: biochemical aspects Baillieres Clin Haematol 1995 8 291 312 10.1016/S0950-3536(05)80269-0 7549064

[B32] Pyke C Kristensen P Ralfkiaer E Eriksen J Danø K The plasminogen activation system in human colon cancer: messenger RNA for the inhibitor PAI-1 is located in endothelial cells in the tumor stroma Canc Res 1991 51 4067 4071 1855221

[B33] Stefansson S Haudenschild CC Lawrence DA Beyond fibrinolysis: the role of plasminogen activator inhibitor-1 and vitronectin in vascular wound healing Trends Cardiovasc Med 1998 8 175 180 10.1016/S1050-1738(98)00003-6 21235930

[B34] Harbeck N Kates RE Schmitt M Gauger K Kiechle M Janicke F Thomassen C Look MP Foekens JA Urokinase-type plasminogen activator and its inhibitor type 1 predict disease outcome and therapy response in primary breast cancer Clin Breast Canc 2004 5 348 352 10.3816/CBC.2004.n.040 15585071

[B35] Duffy MJ Urokinase plasminogen activator and its inhibitor, PAI-1, as prognostic markers in breast cancer: from pilot to level 1 evidence studies Clin Chem 2002 48 1194 1197 12142372

[B36] Berger DH Plasmin/plasminogen system in colorectal cancer World J Surg 2002 26 767 771 10.1007/s00268-002-4050-8 11965442

[B37] Pavey SJ Hawson GA Marsh NA Impact of the fibrinolytic enzyme system on prognosis and survival associated with non-small cell lung carcinoma Blood Coagul Fibrinolysis 2001 12 51 58 10.1097/00001721-200101000-00008 11229827

[B38] Palmirotta R Ferroni P Savonarola A Martini F Ciatti F Laudisi A Sini V Del Monte G Guadagni F Roselli M Prognostic value of pre-surgical plasma PAI-1 (plasminogen activator inhibitor-1) levels in breast cancer Thromb Res 2009 124 403 408 10.1016/j.thromres.2009.02.014 19351570

[B39] Bashar H Urano T Fukuta K Pietraszek MH Hata M Suzuki K Suzuki K Kawabe K Takada Y Takada A Plasminogen activators and plasminogen activator inhibitor 1 in urinary tract cancer Urol Int 1994 52 4 8 10.1159/000282560 8140679

[B40] Millan-Rodriguez F Chechile-Toniolo G Salvador-Bayarri J Palou J Algaba F Vicente-Rodriguez J Primary superficial bladder cancer risk groups according to progression, mortality and recurrence J Urol 2000 164 680 684 10.1016/S0022-5347(05)67280-1 10954628

[B41] Fang H Placencio VR DeClerck YA Protumorigenic activity of plasminogen activator inhibitor-1 through an antiapoptotic function J Natl Canc Inst 2012 104 1470 1484 10.1093/jnci/djs377 PMC3529616 22984202

[B42] Wilkins-Port CE Ye Q Mazurkiewicz JE Higgins PJ TGF-beta1 + EGF-initiated invasive potential in transformed human keratinocytes is coupled to a plasmin/MMP-10/MMP-1-dependent collagen remodeling axis: role for PAI-1 Canc Res 2009 69 4081 4091 10.1158/0008-5472.CAN-09-0043 PMC2962982 19383899

[B43] Freytag J Wilkins-Port CE Higgins CE Carlson JA Noel A Foidart JM Higgins SP Samarakoon R Higgins PJ PAI-1 regulates the invasive phenotype in human cutaneous squamous cell carcinoma J Oncol 2009 2009 963209 20204159 10.1155/2009/963209 PMC2829771

