# Supplementary material for: Matrix metalloproteinase-10 promotes tumor progression through regulation of angiogenic and apoptotic pathways in cervical tumors
Source: BMC Cancer. 2014 May 3;14:310. doi: 10.1186/1471-2407-14-310 (PMC4022983; doi:10.1186/1471-2407-14-310)
Supplement: Additional file 1 — Immunohistochemcal evaluation of tissue microarrays. [file 1471-2407-14-310-S1.docx]

**SUPPLEMENTARY MATERIAL**

# Immunohistochemcal evaluation of tissue microarrays

The TMAs were deparaffinized in xylene, rehydrated using graded percentages of ethanol followed by antigen retrieval using citric acid buffer (pH 6.0, 950C for 20 mins). The slides were treated with 1% hydrogen peroxide in methanol to block endogenous peroxidase activity. Staining for MMP-10 was conducted using rabbit anti-human MMP-10 antibody (ab28206; 1:1000 dilution in blocking buffer, Abcam), biotin-labeled horse anti-rabbit IgG (2 µg/ml in blocking buffer, Vector Laboratories) was used as secondary antibody. Immunoreactive signals were amplified by formation of avidin-biotin peroxidase complexes and visualized using 3, 3'- diaminobenzidine (DAB). Nuclear counterstaining was conducted with hematoxylin. The expression level of MMP-10 was scored by two investigators (GZ and AL) by assigning a proportion score and an intensity score (11). The proportion of positive cells was scored in four grades and represented the estimated proportion of immunoreactive cells (0 = 0% of cells; 1 = 1% to 40%; 2 = 41% to 75% and 3 = 76% to 100%). The intensity was scored and represented the average intensity of positive cells (0 = none; 1 = weak; 2 = intermediate and 3 = strong). The proportion and intensity scores were added to obtain a total MMP-10 staining score, which ranged from 0 to 6. The MMP-10 expression level was determined based on the total MMP-10 staining score as follows: none = 0, low = 1 or 2, moderate = 3 or 4, high = 5 or 6. A third investigator (CJR) reviewed discrepancies and rendered a final score.

# Transfection of small interfering RNA (siRNA)

MMP-10 siRNA -1: sense, CAGGGAAGCUAGACACUGATT; antisense, UCAGUGUCUAGCUUCCCUGTC

MMP-10 siRNA -2: Sense, GAGAAUAUCUGUUCUUUAATT; Antisense, UUAAAGAACAGAUAUUCUCCC.

# Immunoblotting

Total protein from cultured cells was extracted using a RIPA buffer with Halt Protease Inhibitor Cocktail (Thermo Fisher Scientific). Twenty micrograms of total protein (assessed using BCA protein assay) were subjected to SDS-PAGE using 10% acrylamide separating gels. Proteins were transferred to Immobilon-P transfer membranes (Millipore) and stained using a rabbit anti- human MMP-10 antibody (ab28206, dilution 1:2 000; abcam), mouse anti-human GAPDH antibody (sc-47724, dilution 1:5 000, Santa Cruz Biotechnology), mouse anti-human HIF-1α antibody (sc-53546, dilution 1:1 000, Santa Cruz Biotechnology), rabbit anti-human insulin-like growth factor-1 (IGF-1) antibody (sc-9013, dilution 1:1 000, Santa Cruz Biotechnology), rabbit anti-human MMP-2 antibody (sc-10736, dilution 1:1 000, Santa Cruz Biotechnology), rabbit anti-human MMP-9 antibody (ab76003, dilution 1:1 000, abcam), mouse anti-human c-Src tyrosine kinase antibody (#2108, dilution 1:1 000, Cell Signaling Technology) and mouse anti- human PAI-1 antibody (612025, dilution 1:1 000, BD Transduction Laboratories) and appropriate horseradish peroxidase conjugated secondary antibodies (1:5 000). The immune complexes were detected autoradiographically using the ECL Plus Western Blotting Detection System (GE Healthcare). Equal loading was confirmed by GAPDH staining.

# Quantitative reverse transcriptase-PCR

Gene-specific TaqMan primer and probe sets used in this study were Hs00233987_m1 for human

MMP-10 and Hs02758991_g1 for human GAPDH, Mm01168399_m1 for mouse MMP-10 and

Ms99999915_g1 for mouse GAPDH.

# Immunohistochemical (IHC) analysis of xenograft tumors

Paraffin embedded tumor sectioned were deparaffinized in xylene, rehydrated using graded percentages of ethanol. The slide was treated with 1% hydrogen peroxide in methanol to block endogenous peroxidase activity. Staining was conducted using rabbit anti-human MMP-10 (ab28206, dilution 1:1 000, Abcam), goat anti-mouse MMP-10 (sc-26697, dilution 1: 4 000, Santa Cruz Biotechnology), mouse anti-human CD31 (H-3, dilution 1:1 000, Santa Cruz Biotechnology) and rabbit anti-phospho-caspase-3 (#9664, dilution 1:1 000, Cell Signaling Technology). Biotin-labeled horse anti-mouse IgG or rabbit IgG (2 µg/ml in blocking buffer) was used as secondary antibody. Immunoreactive signals were amplified by formation of avidin- biotin peroxidase complexes and visualized using 3, 3'- diaminobenzidine (DAB). Nuclear counterstaining was conducted with hematoxylin. The proportion of MMP-10 positive cells from six randomly selected sections within the xenograft tumors was scored and expressed as a percentage. Microvessel density (MVD) and apoptotic index were quantified from six randomly selected sections and expressed as a percentage of the sum of all positive-stained areas to the total areas (16-18).
