# Supplementary figures and images for: Matrix metalloproteinase-10 promotes tumor progression through regulation of angiogenic and apoptotic pathways in cervical tumors
Source: BMC Cancer. 2014 May 3;14:310. doi: 10.1186/1471-2407-14-310 (PMC4022983; doi:10.1186/1471-2407-14-310)

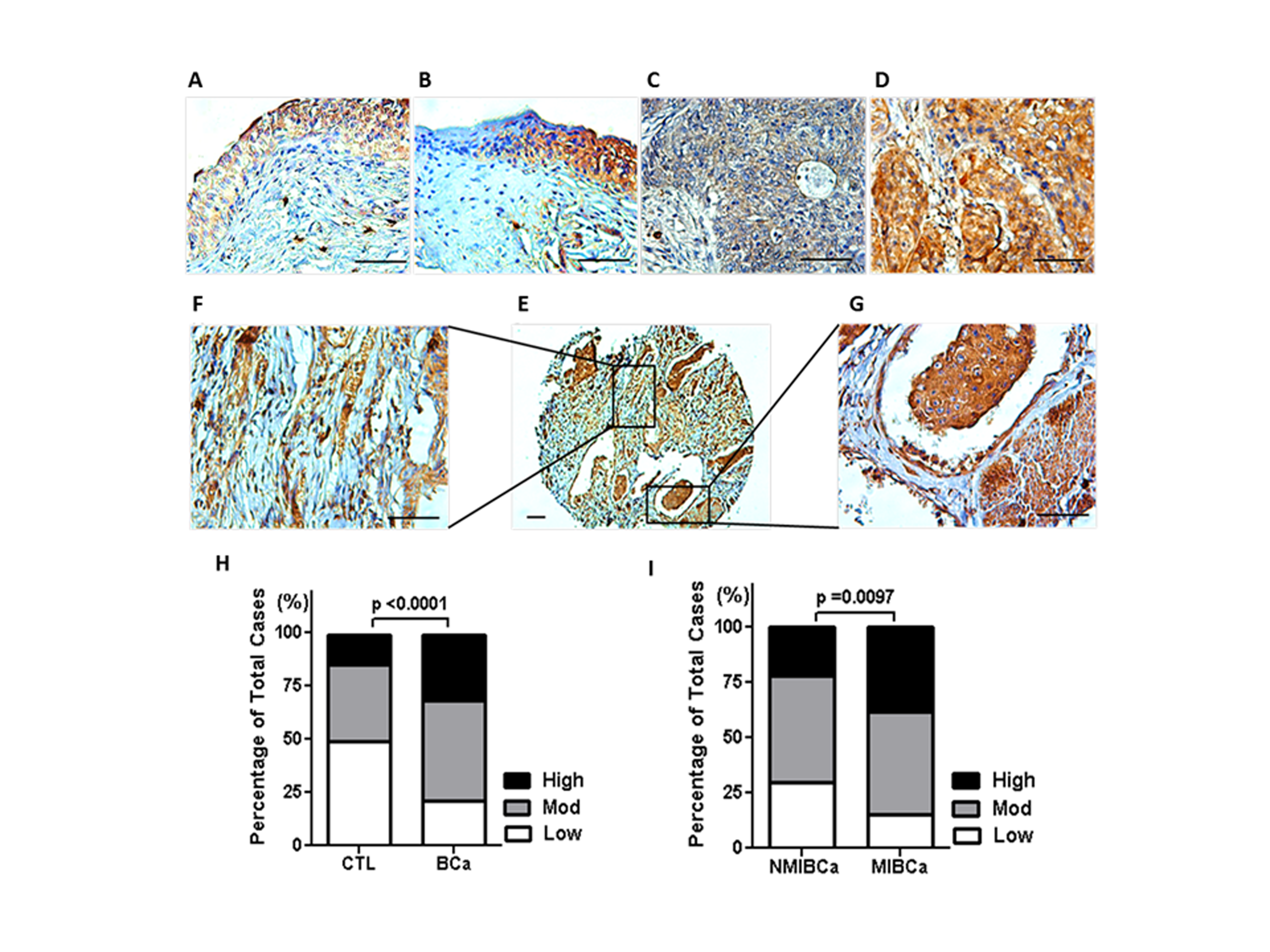

Supplement: Additional file 2: Figure S1 — MMP-10 expression pattern in human bladder cancer. Representative images of normal urothelium (A) and of bladder cancers with absent (B), weak (C), and strong (D) MMP-10 expression (brown) are shown. Representative images of a bladder tissue specimen core on a tissue microarray (E), MMP-10 expression in the tumor stroma (F), and MMP-10 expression in the tumor epithelia (G). Scale bars, 100 μm. H, quantification of MMP-10 expression levels in benign bladder tissue (n = 70) vs. bladder tumor (n = 188). I, quantification of MMP-10 expression levels in non-muscle invasive bladder cancer (NMIBC) vs. muscle invasive bladder cancer (MIBC). Increased MMP-10 expression was noted in bladder tumors compared to benign bladder tissue and the expression levels were noted to be increased in MIBC compared to NMIBC. [file 1471-2407-14-310-S2.tiff]

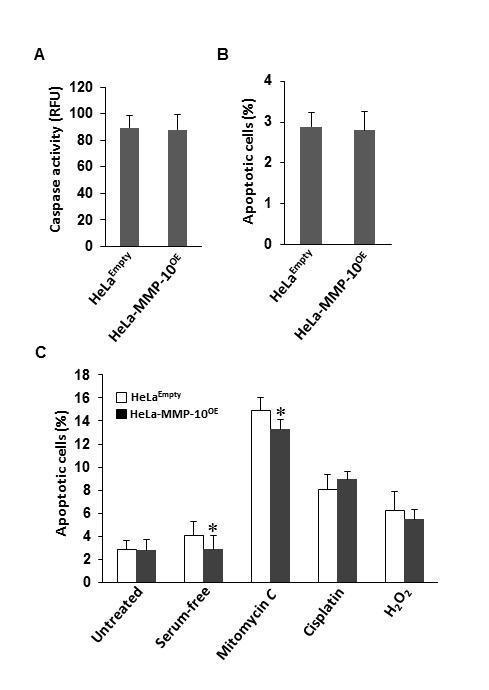

Supplement: Additional file 5: Figure S2 — Apoptotic activity in HeLa cells overexpressing MMP-10. A) Caspase activity assay in HeLaempty and HeLa-MMP-10OE. B) Percentage of cells undergoing apoptosis in HeLaempty and HeLa-MMP-10OE. C) Percentage of HeLaempty and HeLa-MMP-10OE cells undergoing apoptosis after exposure to external stressors. [file 1471-2407-14-310-S5.tiff]
