# Supplementary material for: Matrix metalloproteinase-10 promotes tumor progression through regulation of angiogenic and apoptotic pathways in cervical tumors
Source: BMC Cancer. 2014 May 3;14:310. doi: 10.1186/1471-2407-14-310 (PMC4022983; doi:10.1186/1471-2407-14-310)
Supplement: Additional file 3: Table S1 — RT2 Profiler PCR Array for Angiogenesis (HeLa-MMP-10OE/HeLaEmpty). [file 1471-2407-14-310-S3.docx]

**Additional File 3. RT2 Profiler PCR Array for Angiogenesis.**

| **Table S1. RT^2^ Profiler PCR Array for Angiogenesis**  **(HeLa-MMP-10^OE^/HeLa^Empty^)** | | | |
| --- | --- | --- | --- |
| **Symbol** | **Fold Change** | **Symbol** | **Fold Change** |
| AKT1 | 0.657 | KDR | 1.0813 |
| ANG | 1.1629 | LECT1 | 655.6036 |
| ANGPT1 | 4.2006 | LEP | 0.6823 |
| ANGPT2 | 1.8896 | MDK | 5.1297 |
| ANGPTL4 | 1.2405 | MMP14 | 4.3183 |
| ANPEP | 1.0144 | MMP2 | 27.8939 |
| BAI1 | 1.3467 | MMP9 | 5.064 |
| CCL11 | 1.3467 | NOS3 | 1.8532 |
| CCL2 | 1.5652 | NOTCH4 | 0.8282 |
| CDH5 | 0.5893 | NRP1 | 3.3014 |
| COL18A1 | 0.8443 | NRP2 | 1.2291 |
| COL4A3 | 3.8105 | PDGFA | 0.6481 |
| CTGF | 0.8609 | PECAM1 | 0.9559 |
| CXCL1 | 0.6952 | PF4 | 1.7517 |
| CXCL10 | 1.2586 | PGF | 0.5548 |
| CXCL5 | 1.291 | PLAU | 0.7063 |
| CXCL6 | 1.5973 | PLG | 1.8185 |
| CXCL9 | 4.7485 | PROK2 | 1.3782 |
| EDN1 | 1.1046 | PTGS1 | 0.9317 |
| EFNA1 | 1.6042 | S1PR1 | 0.786 |
| EFNB2 | 1.8636 | SERPINE1 | 1.3467 |
| EGF | 2.4009 | SERPINF1 | 1.0312 |
| ENG | 0.9888 | SPHK1 | 4.4324 |
| EPHB4 | 1.181 | TEK | 1.1074 |
| ERBB2 | 0.5232 | TGFA | 0.7114 |
| F3 | 3.29 | TGFB1 | 0.7397 |
| FGF1 | 23.425 | TGFB2 | 2.7541 |
| FGF2 | 1.1172 | TGFBR1 | 0.9466 |
| FGFR3 | 1.9526 | THBS1 | 3.4178 |
| FIGF | 1.3467 | THBS2 | 1.1069 |
| FLT1 | 0.9286 | TIE1 | 0.5921 |
| FN1 | 1.0498 | TIMP1 | 0.6581 |
| HGF | 0.648 | TIMP2 | 0.7298 |
| HIF1A | 11.0577 | TIMP3 | 1.3823 |
| HPSE | 2.9903 | TNF | 6.6593 |
| ID1 | 5.8766 | TYMP | 0.8117 |
| IFNA1 | 1.7361 | VEGFA | 1.2106 |
| IFNG | 3.4306 | VEGFB | 2.6331 |
| IGF1 | 15.2325 | VEGFC | 0.812 |
| IL1B | 20.4366 | ACTB | 0.8909 |
| IL6 | 0.6599 | B2M | 1.2181 |
| IL8 | 1.1811 | GAPDH | 0.7551 |
| ITGAV | 1.4379 | HPRT1 | 1.1044 |
| ITGB3 | 0.8057 | RPLP0 | 1.1049 |
| JAG1 | 1.0781 | HGDC | 4.6241 |
