# Supplementary material for: Matrix metalloproteinase-10 promotes tumor progression through regulation of angiogenic and apoptotic pathways in cervical tumors
Source: BMC Cancer. 2014 May 3;14:310. doi: 10.1186/1471-2407-14-310 (PMC4022983; doi:10.1186/1471-2407-14-310)
Supplement: Additional file 4: Table S2 — RT2 Profiler PCR Array for Metastasis (HeLa-MMP-10OE/HeLaEmpty). [file 1471-2407-14-310-S4.docx]

**ADDITIONAL FILE 4. RT^2^ Profiler PCR Array for Metastasis.**

| **Table S2. RT^2^ Profiler PCR Array for Metastasis**  **(HeLa-MMP-10^OE^/HeLa^Empty^)** | | | |
| --- | --- | --- | --- |
| **Symbol** | **Fold Change** | **Symbol** | **Fold Change** |
| APC | 0.657 | MGAT5 | 1.0813 |
| BRMS1 | 1.1629 | MMP10 | 655.6036 |
| CCL7 | 4.2006 | MMP11 | 0.6823 |
| CD44 | 1.8896 | MMP13 | 5.1297 |
| CD82 | 1.2405 | MMP2 | 4.3183 |
| CDH1 | 1.0144 | MMP3 | 27.8939 |
| CDH11 | 1.3467 | MMP7 | 5.064 |
| CDH6 | 1.3467 | MMP9 | 1.8532 |
| CDKN2A | 1.5652 | MTA1 | 0.8282 |
| CHD4 | 0.5893 | MTSS1 | 3.3014 |
| COL4A2 | 0.8443 | MYC | 1.2291 |
| CST7 | 3.8105 | MYCL1 | 0.6481 |
| CTBP1 | 0.8609 | NF2 | 0.9559 |
| CTNNA1 | 0.6952 | NME1 | 1.7517 |
| CTSK | 1.2586 | NME4 | 0.5548 |
| CTSL1 | 1.291 | NR4A3 | 0.7063 |
| CXCL12 | 1.5973 | PLAUR | 1.8185 |
| CXCR2 | 4.7485 | PNN | 1.3782 |
| CXCR4 | 1.1046 | PTEN | 0.9317 |
| DENR | 1.6042 | RB1 | 0.786 |
| EPHB2 | 1.8636 | RORB | 1.3467 |
| ETV4 | 2.4009 | RPSA | 1.0312 |
| EWSR1 | 0.9888 | SERPINE1 | 4.4324 |
| FAT1 | 1.181 | SET | 1.1074 |
| FGFR4 | 0.5232 | SMAD2 | 0.7114 |
| FLT4 | 3.29 | SMAD4 | 0.7397 |
| FN1 | 23.425 | SRC | 2.7541 |
| FXYD5 | 1.1172 | SSTR2 | 0.9466 |
| GNRH1 | 1.9526 | SYK | 3.4178 |
| HGF | 1.3467 | TCF20 | 1.1069 |
| HPSE | 0.9286 | TGFB1 | 0.5921 |
| HRAS | 1.0498 | TIMP2 | 0.6581 |
| HTATIP2 | 0.648 | TIMP3 | 0.7298 |
| IGF1 | 11.0577 | TIMP4 | 1.3823 |
| IL18 | 2.9903 | TNFSF10 | 6.6593 |
| IL1B | 5.8766 | TP53 | 0.8117 |
| ITGA7 | 1.7361 | TRPM1 | 1.2106 |
| ITGB3 | 3.4306 | TSHR | 2.6331 |
| KISS1 | 15.2325 | VEGFA | 0.812 |
| KISS1R | 20.4366 | ACTB | 0.8909 |
| KRAS | 0.6599 | B2M | 1.2181 |
| MCAM | 1.1811 | GAPDH | 0.7551 |
| MDM2 | 1.4379 | HPRT1 | 1.1044 |
| MET | 0.8057 | RPLP0 | 1.1049 |
| METAP2 | 1.0781 | HGDC | 4.6241 |
